# Facial soft tissue changes following isolated bilateral sagittal split osteotomy for mandibular advancement and setback, a review

**DOI:** 10.1186/s12903-026-07838-1

**Published:** 2026-02-15

**Authors:** Xhenisera Hallulli, Mats Sjöström

**Affiliations:** Department of Odontology, Umeå University, Umea, Sweden

**Keywords:** Bilateral Sagittal Split Osteotomy, Mandibular Advancement, Mandibular Setback, Soft Tissue Changes, Hard-to-Soft Tissue Ratio

## Abstract

This review evaluated literature on soft tissue changes in patients following bilateral sagittal split osteotomy (BSSO) for mandibular advancement or setback. Twenty published articles were identified for further analysis. The included articles related to patient cohorts ranging between 12 and 109 participants, the age range of 14–68 years, with a majority of female patients, and follow-up periods in the range of 6–114 months. The primary outcomes were soft tissue changes in the cephalometric soft tissue points; pogonion (Pg'); menton (Me'); labrale inferius (Li); and mentolabial fold (B'). Soft-to-hard tissue ratios varied widely across both conventional and alternative mandibular procedures, with higher ratios observed for advancement in the conventional group. For example, at pogonion (Pg’) in advancement cases, ratios ranged from 80 – 133%. This study highlights the complexity of soft tissue changes following bilateral sagittal split osteotomy (BSSO). The variability seen in outcomes underscores the need for longer follow-up periods and surgery after skeletal growth has waned. Despite the valuable insights gained from the literature, considerable variability underscores the influence of skeletal relapse, age, and fixation type. Standardized long-term 3D studies are warranted to refine predictive models when isolated BSSO is performed.

## Background

Orthognathic surgery aims to (a) correct severe types of dentofacial skeletal deformities and malocclusion that cannot be adequately addressed by orthodontic treatment alone, (b) attain a balanced facial harmony and esthetics, (c) optimize temporomandibular joint function, and (d) improve neuromuscular function. In most cases, orthodontic treatment is required both before and after orthognathic surgery. Facial tissues are supported by, among other structures, the maxilla and mandible, which means that orthognathic surgery usually alters the facial appearance. The biological basis of these changes parallels the remodeling response seen during orthodontic tooth movement, where controlled mechanical or surgical forces trigger inflammation-mediated bone and soft-tissue adaptation. These forces induce aseptic periodontal inflammation characterized by cytokine release and osteoclastic–osteoblastic activity [1], mechanisms that also underlie the postsurgical remodeling phase following orthognathic procedures.

The facial appearance is a summation of the facial skeleton, dentition and soft tissue. Assessment of facial appearance can be used for evaluation of treatment [2]. Facial asymmetries can originate from one or more skeletal structures, dental asymmetry due to early tooth loss or missing teeth, and soft tissue asymmetries attributed to abnormal muscle function. Functional asymmetry may result from mandibular deviation due to tooth interference [3]. The anatomical variations mentioned above frequently presents symptoms that include headaches, snoring, sleep apnea, diminished chewing ability, respiratory issues, speech difficulties, and temporomandibular joint disorders in the individual patient [4], pp.220–223).

During preoperative information, prior to orthognathic surgery, caution should be exercised regarding the postoperative aesthetic outcome as predictions are uncertain [5, 6].

**Fig. 1:**
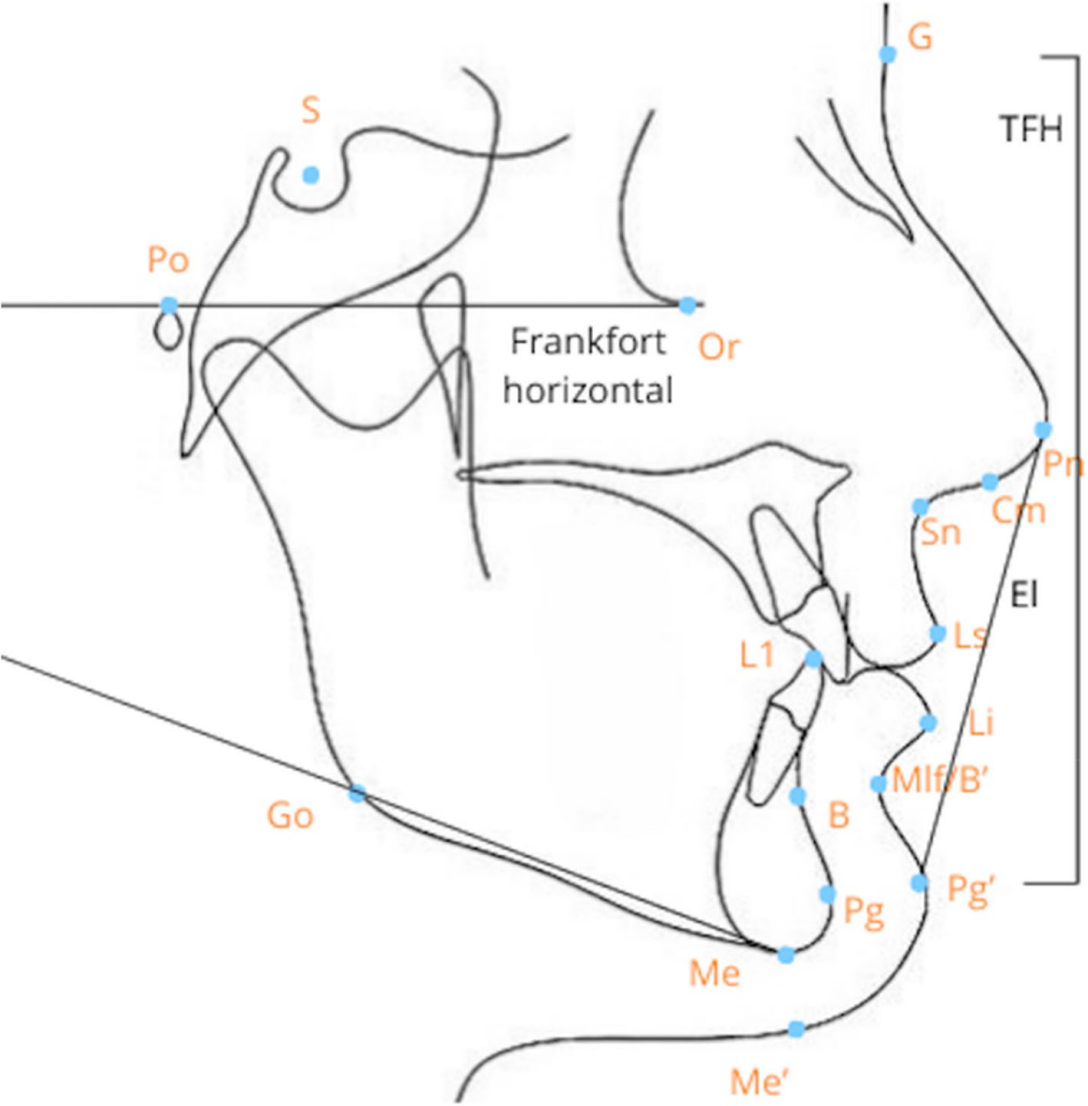
Dental, skeletal and soft tissue landmarks used in clinical and radiographic examinations. Profile with Dental, skeletal and soft tissue landmarks. Abbreviations are described in Table 1. This figure has been adapted from Uysal et al. [7] and modified by the authors of the present study

All the functions mentioned above should be achieved with interventions that result in stable long-term results. During patient evaluation, measurements of the face, jaws and intraoral status are documented. The occlusion is evaluated in sagittal, vertical and transverse dimension using plaster casts or intraoral scanning transformed into 3D images. Descriptions of facial appearance are often made with facial convexity and nasolabial angle. Figure 1 illustrates dental, skeletal and soft tissue landmarks used in clinical and radiographic examinations. Table 1 describes definitions of dental, skeletal and soft tissue landmarks and angles.

**Table 1:**
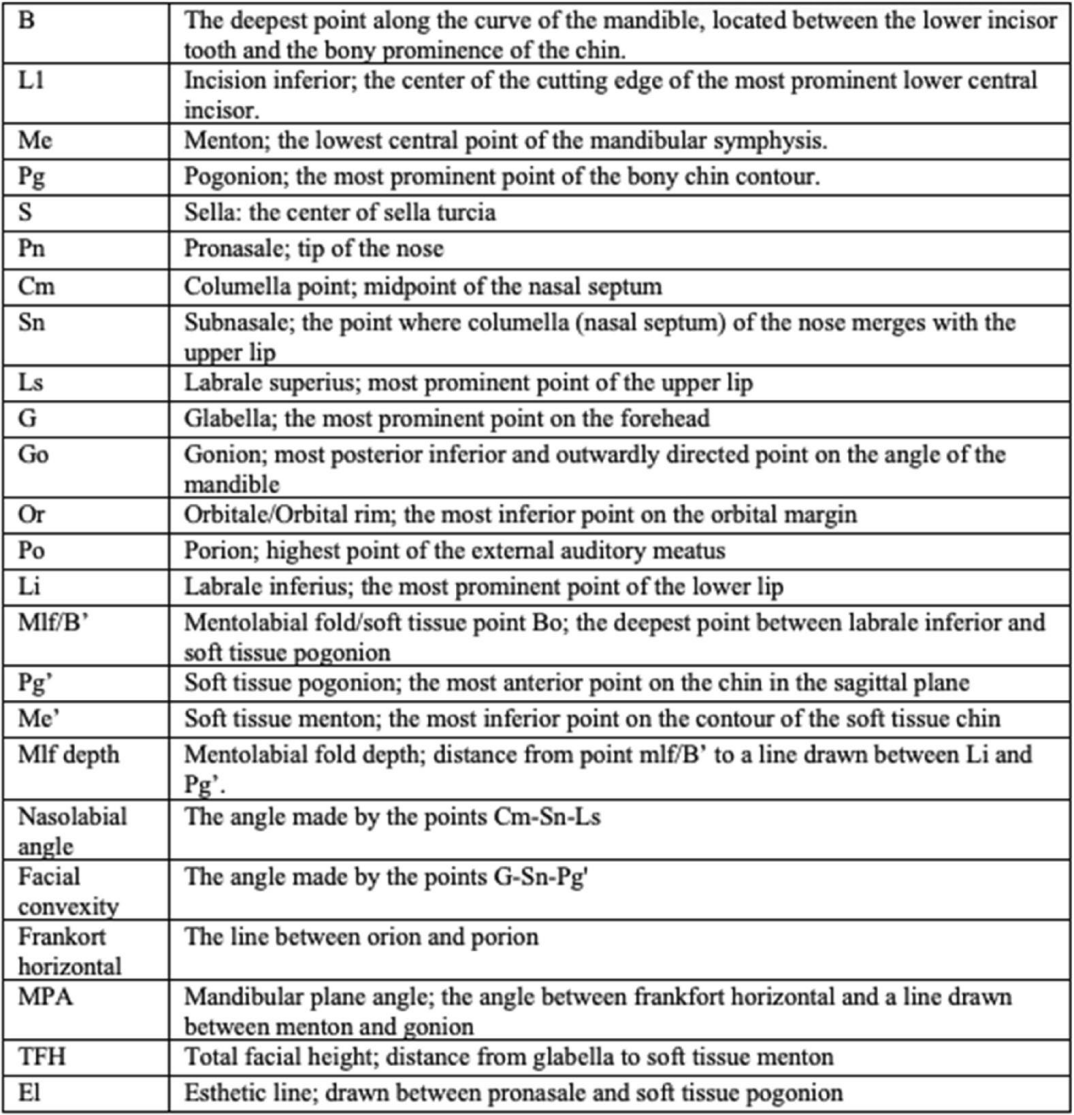
Dental, skeletal and soft tissue landmarks made for descriptions of facial appearance

Dental, skeletal and soft tissue landmarks made for descriptions of facial appearance

The table has been adapted from DiPietro and Moergeli [8], and Mobarak et al. [9] and modified by the authors of the present study

Radiographic examinations are used for cephalometric measurements, which can be obtained from 2D lateral images or advanced 3D imaging using computed tomography (CT) scans [10], pp.417–427). CT scans are particularly effective for assessing the 3D changes in both hard and soft tissues after orthognathic surgery [11].

Statistically significant distinctions have been observed between 2 and 3D cephalometric analyses regarding certain angle measurements. However, an individual measurement can have less value when assessing the symmetry of the entire face and should not influence the treatment decision-making [12]. A 3D evaluation is superior to 2D surgical planning of facial asymmetry patients, and though virtual surgical planning (VSP) is a valuable tool, measurements for skeletal repositioning should be carried out clinically [13], pp.435–442). Cephalometric values alone are insufficient for assessing different esthetic facial components and should, therefore, be considered solely as a supplement to clinical facial analysis [14].

The Frankfort horizontal plane serves as a reference plane to standardize skull analysis. It has been established by connecting the highest point of the bony external auditory meatus (anatomical porion, PoA) to the lowest point on the bony left orbital rim (Orbitale, Or), [15]. The mandibular plane angle (MPA) is the angle between the mandibular plane (which is a line drawn from the menton to the gonion) and the Frankfort horizontal plane [8]. It is used to assess the relationship between the mandible and the skull, classifying individuals into hyperdivergent, average, and hypodivergent categories.

The most-commonly applied orthognathic surgical procedure in the mandible is the Bilateral Sagittal Split Osteotomy (BSSO). Indications for BSSO include skeletal mandibular retrognathia, prognathia or mandibular asymmetries and therefore used both in mandibular advancement and mandibular setback surgery [16, 17]. After mandibular movements, significant changes in facial soft tissues in relation to the three dimensions of space (vertical, sagittal and transverse) are recognized. The changes seen relate to the ratio of movement between soft and hard tissues,soft tissue volume changes; changes in the mentolabial and nasolabial angle; and changes of different hard and soft tissue landmarks in relation to each other [18].

Mandibular setback surgery affects the convexity of the facial profile, as well as the nasolabial angle and mentolabial fold [9]. Mandibular advancement surgery results in changes in the lower facial height, facial convexity, depth of the mentolabial fold, as well as changes to the lower lip [19].

Factors such as gender, the extent of movement of the mandible and skeletal stability affect the outcome of soft tissue changes [9]. The accuracy of soft tissue predictions is affected by gender, race, mean ratios of hard to soft tissue changes, and the extent of skeletal repositioning [20].

Wire fixation has been associated with a higher rate of skeletal relapse compared to rigid fixation methods. Since skeletal stability influences soft tissue adaptation, the choice of fixation technique can impact soft tissue outcomes [21, 22]. Correct condylar positioning is vital for stable long-term results. Condylar displacement can cause malocclusion, skeletal relapse and condylar resorption [23]

Soft tissue changes are more predictable after larger setbacks (> 9 mm), as compared with smaller setbacks (3–6 mm), while vertical displacements are less predictable than horizontal repositioning [9].

In addition, soft tissue changes after BSSO are influenced by the mandibular plane angle (MPA). Patients with a low MPA (mean 77.8) show greater posterior repositioning of the labrale superius (Ls), an increase in the nasolabial angle, and a more-noticeable increase in the distance of labrale inferius to the esthetic line, as compared to patients with high (mean 70.5) and medium (mean 74.0) MPAs. In cases of skeletal relapse, the soft tissue chin and mentolabial fold preserve their relationship (approximately 1:1 ratio). As previously mentioned, it has been suggested that skeletal relapse for various orthognathic procedures be considered when predicting soft tissue outcomes [24].

The Swedish Council on Health Technology Assessment (SBU) and Database of Uncertainties about the Effects of Treatments (UK DUETs) have identified gaps in the knowledge related to specific areas of oral and maxillofacial surgery. This gap is due to the absence of systematic reviews and reflects the fact that existing reviews highlight uncertainties regarding treatment outcomes. One area of uncertainty is related to the soft tissue changes following isolated bilateral sagittal split osteotomy [25].

The BSSO is the most performed orthognathic surgical procedure in Sweden [26]. Although several studies have described skeletal outcomes of BSSO, the translation of these skeletal movements to predictable soft tissue responses remains uncertain. This inconsistency prompted a focused review of current literature to clarify these relationships.

Therefore, the aim of this study is to review the existing literature on soft tissue changes after isolated BSSO surgery, to address the knowledge deficiencies. To strengthen the rationale, the review is explicitly limited to isolated BSSO, to avoid the confounding influence of maxillary surgery and genioplasty, thereby allowing for more precise conclusions regarding the effects of BSSO. This focus enhances the scientific validity of the study and the possibility to address the identified knowledge deficiencies.

## Material and methods

### Search strategy

To evaluate the effects of isolated BSSO surgery on patients’ facial soft tissues, literature searches were conducted in the PubMed and CINAHL databases.

The following search string was used to search the databases:

(((((("orthognathic surgery"[MeSH Terms]) OR ("orthognathic surgical procedures"[MeSH Terms])) OR ("osteotomy, sagittal split ramus"[MeSH Terms])) OR (bilateral sagittal split osteotomy)) OR (mandibular advancement)) OR (mandibular setback)) AND (soft tissue).

### Inclusion/exclusion

The following inclusion criteria were applied:

- Original research/original articles that are peer reviewed and written in English language.
- No limitations as to year of publication.
- A BSSO is performed on human patients with asymmetry or a prognathic or retrognathic mandible (with or without vertical discrepancies).
- No limitation regarding the patient age when surgery was performed.
- Two-dimensional radiographic examination.
- A follow-up period ≥ 6 months.

The following exclusion criteria were applied:

- Patients underwent revision surgery.
- Patients underwent bimaxillary surgeries.
- Patients underwent genioplasty.
- Use of alternative surgical techniques for the mandible, such as “intraoral vertical ramus osteotomy” and “extraoral ramus osteotomy”.
- Patients with cleft lip and palate, and facial skeletal discrepancies associated with various syndromes.

### Screening process

**Fig. 2:**
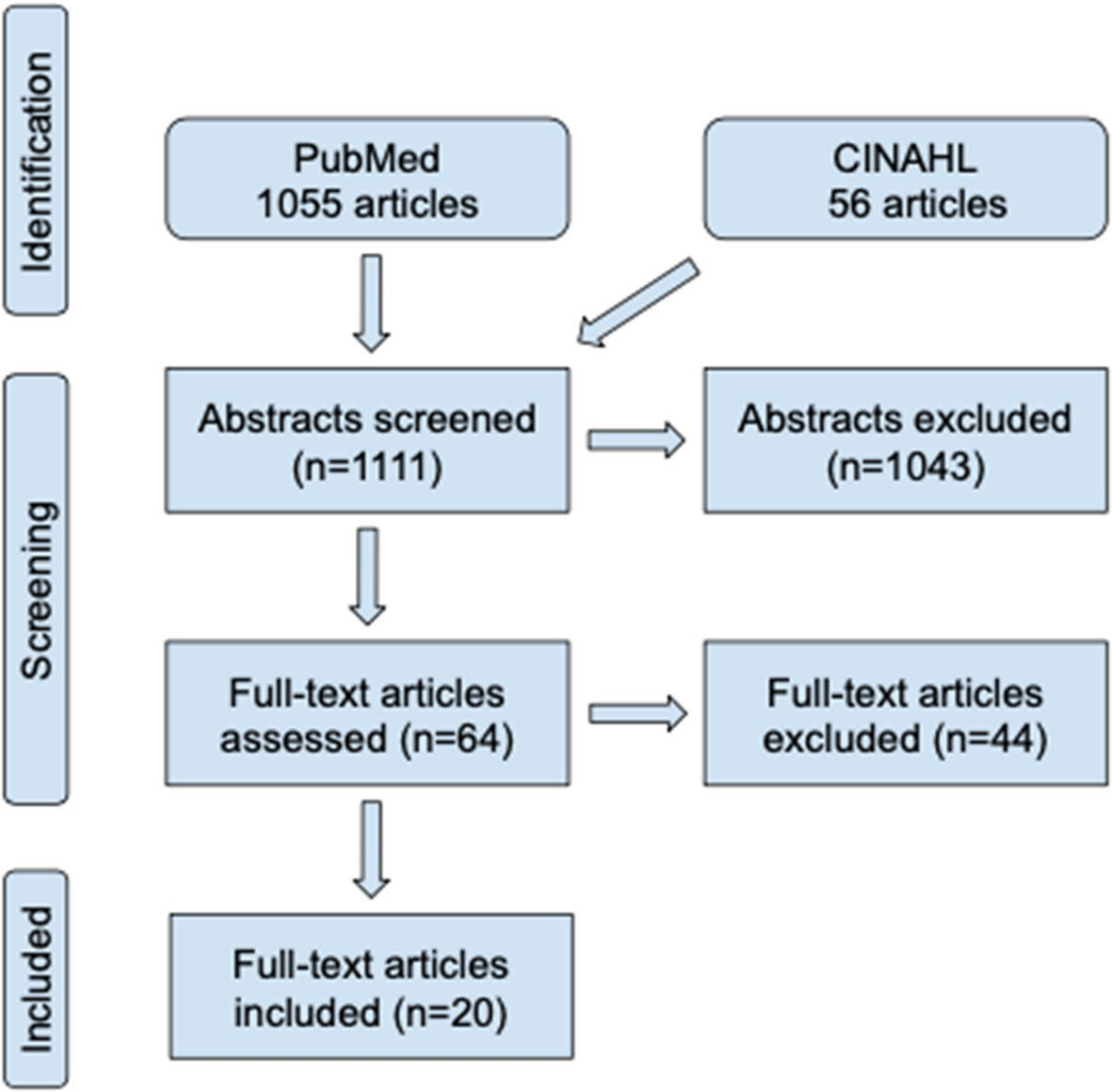
Flowchart

A search performed on October 7, 2024, generated 1055 articles in PubMed and 56 in CINAHL, using the filters “english”, “human” and “peer reviewed”. The titles and abstracts were screened, and additional relevant studies were identified through suggestions for “similar articles” and reference lists. In total, 64 articles were read in full text. After applying the predefined inclusion and exclusion criteria listed above, 20 articles were selected for inclusion, as illustrated in Fig. 2*.*

The selected articles were initially categorized into four groups based on whether the patients were skeletally growing or non-growing at the time of surgery, as well as the duration of follow-up (6 months or > 1 year). Given that two out of the four groups each comprised only 2–3 articles, the findings derived from these groups were deemed to be irrelevant. Therefore, the articles were reorganized into two groups, based on the type of surgery: (a) BSSO for mandibular advancement; and (b) BSSO for mandibular setback.

## Results

This study reviewed 20 articles published between 1987 and 2019 (Fig. 2). Included studies reported between 12 and 109 participants with mean ages ranging from 17,8 to 36 years. Most studies (89%) had a higher proportion of female patients. Follow-up periods varied between 6 and 114 months, with the majority having a follow-up period of 12–36 months.

The results included conventional ratios of hard to soft tissue changes, with cephalometric values taken before surgery (T0) and at least 6 months post-surgery (T2). In addition, some studies presented an alternative ratio for hard to soft tissue changes by comparing the soft tissue changes over time (T0-T2) with the surgical hard tissue changes immediately after surgery (T0-T1):

Conventional ratio: Net soft tissue change (T0-T2)/Net hard tissue change (T0-T2) × 100;

Alternative ratio: Net soft tissue change (T0-T2)/Surgical hard tissue change (T0-T1) × 100.

Of the 20 studies included, 18 provided information on ratios, with 10 focusing on mandibular advancement and 8 on mandibular setback through BSSO. While the total number of studies remained at 20, the types of ratios used varies: conventional ratio was presented in 10 studies, alternative ratio in one study, both ratios in four studies, and one study did not specify.

This resulted in a set of conventional and alternative ratios for both mandibular advancement and setback procedures. In general, higher ratios were typically observed in advancement procedures compared to setback procedures, in the conventional groups. The full range of values are presented in Tables 2 and 3.

**Table 2:**
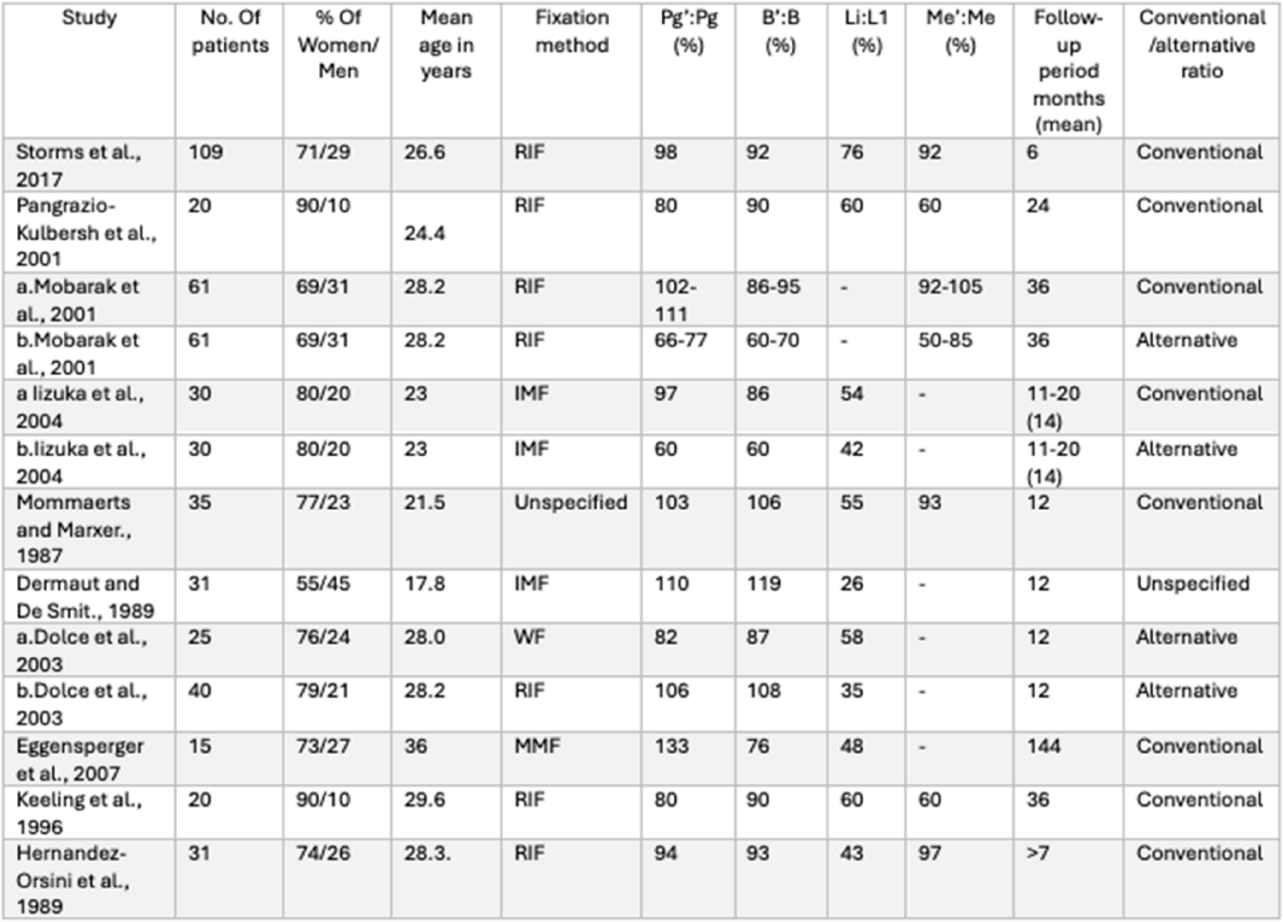
Summary of articles describing the ratios of hard tissue to soft tissue changes following mandibular advancement through BSSO [9, 21, 22, 24, 27–33]

The presented ratios are for the following landmarks: hard and soft tissue pogonion (Pg/Pg'), point B and mentolabial fold (B/B'), inferior incisor and labrale inferior (L1/Li), and hard and soft tissue menton (Me/Me'). All ratios are measured in the anteroposterior direction, except for Me/Me', which is generally measured vertically in most studies, although some studies did not specify the direction. Studies listed twice are explained by the different fixation techniques used: RIF, Rigid internal fixation; IMP, Intermaxillary fixation; and MMF, Maxillomandibular fixation

**Table 3:**
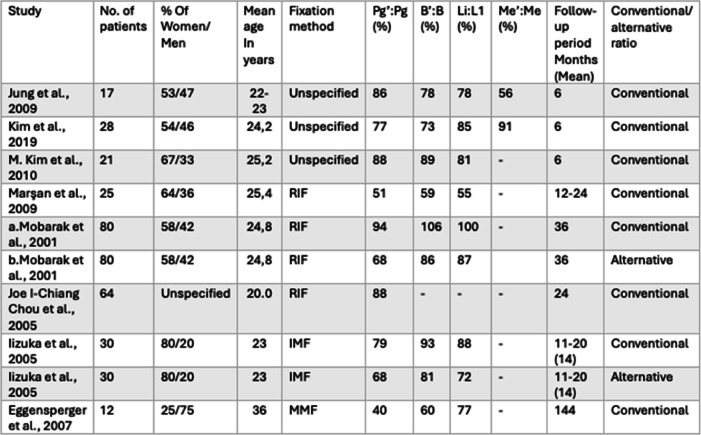
Summary of articles describing the ratios of hard to soft tissue changes following mandibular setback through BSSO [9, 24, 28, 34–39]

The ratios presented are for the following landmarks: hard and soft tissue pogonion (Pg/Pg'), point B and mentolabial fold (B/B'), inferior incisor and labrale inferior (L1/Li), and hard and soft tissue menton (Me/Me'). All of the ratios are measured in the anteroposterior direction, except for Me/Me', which is generally measured vertically in most studies, although some studies did not specify the direction. Studies listed twice are explained by the different fixation techniques used: RIF, Rigid internal fixation; IMP, Intermaxillary fixation; and MMF, Maxillomandibular fixation

## Discussion

It is widely recognized that BSSO primarily impacts the lower lip and the soft tissue of the chin [40]. In the present study, we have evaluated the soft tissue changes for solely BSSO procedures and found an extensive variability in soft tissue ratios. In the present study, following cephalometric soft tissue points were evaluated: labrale inferius (Li), mentolabial fold (B’), soft tissue pogonion (Pg’) and soft tissue menton (Me’). In addition, nasolabial angle, the depth and angle of the mentolabial fold, and facial convexity were analyzed.

In individuals with class 3 malocclusion, compensatory tooth positions, such as proclined upper incisors and retroclined lower incisors, are frequently observed. These altered positions improve functionality and conceal the dentofacial discrepancy. Adequate decompensation of the mandibular incisors is crucial to the success of the procedure and ensuring long-term stability [41, 42]

### Main findings

The present study shows that after mandibular setback surgery in patients with angle class III relations, the common soft tissue changes include increased facial convexity and a deeper mentolabial fold. These changes bring the patient’s profile closer to the esthetic norms. On the other hand, mandibular advancement surgery for class II patients results in forward movement of the chin (Pg’ and B’), reduced profile concavity, and an increase in facial height.

### Influence of skeletal pattern

Differences in soft tissue outcomes and specific angle measurements following BSSO were observed when patients were grouped by skeletal angle classifications, such as hypodivergent, average, and hyperdivergent. For instance, the hard/soft tissue change ratio at the pogonion after mandibular advancement varied for different mandibular plane angle groups, ranging from 102–111% for the conventional ratio and 66–77% for the alternative ratio [24]. These findings suggest that soft tissue changes following BSSO should be calculated separately for each skeletal angle type.

Of the articles included in the present study, five classified patients based on the mandibular plane angle [24, 33, 35, 37, 40]. The studies noted variations in soft tissue outcomes across the different angle groups, although most of these differences were not statistically significant. One possible explanation for this lack of significance is the small sample sizes, highlighting the need for studies with larger patient cohorts.

### Fixation method and relapse

A common theme in the selected studies is the impact of skeletal relapse on the final esthetic outcome. Many studies have noted that the initial soft tissue changes observed postoperatively might not be entirely maintained, due to skeletal shifts that occur over time. Studies that follow patients over a long period of time often report that the soft tissue profiles continue to adapt to skeletal changes. The articles indicated ongoing soft tissue adjustments for at least 1 year following BSSO, suggesting that additional surgical procedures should be postponed until a thorough evaluation of the actual final soft tissue outcomes is completed. This is further reinforced by the findings of another article [43].

Two studies reported both conventional and alternative ratios for mandibular setback and advancement. The findings revealed that the alternative ratios were considerably lower than the conventional ratios for all the reported landmarks. This indicates that the hard/soft tissue ratios decrease when skeletal relapse is considered. However, since skeletal relapse can vary considerably from one case to another, it becomes difficult to draw a clear and definitive conclusion.

Several studies have examined different fixation methods, comparing rigid internal fixation to wire fixation. Two studies found that rigid fixation provided greater stability, while wire fixation was associated with a 30% rate of skeletal relapse [21, 22].

Short-term follow-up comparing RIF to WF demonstrated that RIF was associated with higher ratios in Pg’:Pg and Li:L1. In the long-term follow-up, RIF also showed higher ratios in Mlf:B’ and Pg’:Pg compared to WF. However, the differences were not significant. It should be noted that the study included only 12 articles, and for the long-term data, there were fewer than two studies per group. Although some differences were observed, they were small [44]. One can speculate about the impact of soft tissue changes versus the impact of post-operative skeletal changes.

### Growth and age considerations

Between the ages of 13 and 16, there is a significant age-related change in dentofacial growth, especially in the lower jaw, with a noticeable growth spurt more distinct in males. Residual growth of the mandible, particularly in young adult males, must be considered during orthognathic surgery [45]. The findings of the present study are based on articles that concern patients of minimum age of 14 years, which means that some of the study subjects may not have completed skeletal growth of the mandible. This raises concerns about the remaining growth of the mandible during this crucial growth spurt. Specifically, the growth spurt between 13 and 16 years of age could impact the outcomes of surgery, as the mandible may still be growing after the procedure, potentially influencing long-term results.

In some cases, early intervention has been associated with better quality of life in growing individuals, although there is a possibility that secondary surgical procedure may be required [46].

### Limitations of current evidence and the present study

The absence of meta-analysis precludes pooled estimation of effect sizes.

3D analysis was not included in the present study. The application of 3D measurements to the evaluation of soft tissue changes following orthognathic surgery is expanding, particularly in the context of bimaxillary procedures. However, our study specifically concentrated on soft tissue changes following isolated mandibular surgery. Only a limited number of studies have employed 3D techniques to evaluate soft tissue changes before and after isolated BSSO surgery. 2D cephalometry has limited accuracy due to errors in landmark identification, magnification, and patient positioning. Curved or dental landmarks are especially prone to mistakes [47]. CBCT provides more accurate measurements by avoiding magnification, overlap, and distortion problems seen in 2D imaging [48]. While 3D offers superior volumetric analysis, the lack of standardized measurement points and methodologies makes meta-analysis and direct comparison with the vast 2D literature currently unfeasible. Consequently, articles using 3D measurments were excluded. However, 3D analysis represents a potential area for future standardization of soft tissue evaluation.

No assessment of bias was performed. Not evaluating the quality of the studies included makes the results less reliable and lowers confidence in the conclusions.

### Sources of heterogenity and clinical implications

The wide range of results observed in the included studies reflect substantial variability. This heterogeneity—arising from differences in study design, such as variations in patient cohorts, age, MPA, skeletal relapse, and follow-up period—makes it difficult to draw firm conclusions. It also highlights the inherent unpredictability of soft tissue outcomes and the likely influence of additional, unaccounted-for factors. The present study had a higher proportion of female patients. There is a slight overweight of women performing orthognathic surgery according to Sjöström et al. [26]. The studies included in the analysis were conducted across multiple countries. Gender distribution for each country among individuals undergoing orthognathic surgery was not evaluated. This shortcoming limits the generalizability of the findings.

A 1:1 ratio for the chin is often cited in the literature [24, 27, 31, 40, 44]. However, some studies that have come to this conclusion vary in terms of their methodologies. Some involve genioplasty, others focus on bimaxillary surgery, and most of them vary with respect to the follow-up period, yet they are often presented in the same table. It is essential to have clear definitions and to ensure that only similar studies are compared. In the present study, certain articles were included in which the patients either received bimaxillary surgery or isolated BSSO. The results shown in Table 3 and 4 are from the groups that did not undergo bimaxillary surgery and therefore met the inclusion criteria. The present review of isolated BSSO studies reveals significant variability, challenging the predictability of the assumption of a 1:1 ratio of the chin.

In summary, the accuracy of predictions can be significantly affected by the considerable variability of factors such as the thickness, tonicity, posture, and length of the soft tissue covering. These highly variable elements may lead to inconsistencies, highlighting the challenges associated with predicting soft tissue outcomes [49]. Future research into adjunctive therapies, such as the use of advanced biomaterial scaffolds [50], could help to modulate the soft tissue environment and improve the predictability of healing. The application of such innovative biomaterials during or following orthognathic surgery could potentially provide a more favourable and controllable biological foundation for both hard and soft tissue healing, thereby improving overall aesthetic and functional outcomes.

## Conclusion

The study of soft tissue changes following solely BSSO surgery is complex, influenced by various factors, such as age, gender, skeletal relapse, mandibular plane angle, and the method of fixation. The variability in soft tissue outcomes emphasizes the importance of longer follow-up periods, ideally extending beyond 5 years, to accurately assess true stability. Furthermore, it is crucial that surgery is performed after skeletal growth has declined. Although the literature reviewed provides valuable insights, further research is needed to deepen our understanding of soft tissue responses and to develop more reliable predictive models. Thus, drawing definitive conclusions about soft tissue changes after BSSO remains challenging. Therefore, future studies should emplow 3D longitudinal multicentric designs and AI-based predictive modeling.

## Data Availability

The datasets generated and/or analyzed during the current study are available in the [PubMed] and [CINAHL] repository, [https://pubmed.ncbi.nlm.nih.gov/], [https://web.p.ebscohost.com/ehost/search/advanced?vid=0&sid=1ac60b94-d298-428e-93d0-abc7503ac5ff%40redis].

## Abbreviations

2D: Two-dimensional
3D: Three-dimensional
B: Point B
BSSO: Bilateral sagittal split osteotomy
CLP: Cleft lip and palate
Cm: Columella point
CT: Computed tomography
G: Glabella
IMF: Intermaxillary fixation
L1: Incision inferior
Li: Labrale inferius
Ls: Labrale superius
Me: Menton
Mlf: Mentolabial fold
Mm: Millimeters
MMF: Maxillomandibular fixation
MPA: Mandibular plane angle
Or: Orbital rim
Pg: Pogonion
PoA: Anatomical porion
RIF: Rigid internal fixation
SBU: The Swedish Council on Health Technology Assessment
Sn: Subnasale
TFH: Total facial height
UK DUETs: Database of Uncertainties about the Effects of Treatments
VSP: Virtual surgical planning
WF: Wire fixation
